# The survival benefit of neoadjuvant chemotherapy and pCR among patients with advanced stage triple negative breast cancer

**DOI:** 10.18632/oncotarget.22521

**Published:** 2017-11-20

**Authors:** Tithi Biswas, Jimmy T. Efird, Shreya Prasad, Charulata Jindal, Paul R. Walker

**Affiliations:** ^1^ Department of Radiation Oncology, University Hospitals, Case Western Reserve University, Cleveland, OH, USA; ^2^ Centre for Clinical Epidemiology and Biostatistics, School of Medicine and Public Health, University of Newcastle, Newcastle, NSW, Australia; ^3^ Department of Internal Medicine, North Shore-Long Island Jewish Medical Center, Manhasset, NY, USA; ^4^ Division of Hematology/Oncology, Vidant Health Cancer Care, Greenville, NC, USA

**Keywords:** adjuvant chemotherapy, disease free survival, neoadjuvant chemotherapy, overall survival, triple negative breast cancer

## Abstract

Triple negative breast cancer (TNBC) is an aggressive subtype that accounts for 15-20% of cases, with a higher incidence of relapse/death. Even with adjuvant chemotherapy, the 5 year distant metastasis-free survival rate remains low. A total of 452 tumor registry patients with TNBC and no evidence of metastatic disease were identified over the period of 1996-2011. The median age and follow-up time were 51 (range=21-88) and 3.9 (range=0.14-14) years. Approximately 75% of patients with stage III disease received neoadjuvant chemotherapy (NACT) compared with 47% for stage II. Patients with stage I disease predominantly received adjuvant chemotherapy (ACT). Among those who underwent NACT (n=202), 33% had a pathological complete response (pCR). Overall (OS) and disease-free (DFS) survival were significantly longer among patients achieving pCR (versus residual disease) following NACT (OS: all patients *P*<0.0001, stage II *P*<0.0001, stage III *P*=0.0062; DFS: all patients *P*<0.0001, stage II *P*=0.0011, stage III *P*=0.015). ACT was not associated with improved OS or DFS for stage III disease. Adjustment for age, chemotherapy, health insurance type, lymphovascular invasion, race, radiation, and surgery did not alter our results. These findings suggest that pCR following NACT is associated with improved survival among patients with TNBC, independent of diagnostic stage.

## INTRODUCTION

Triple negative breast cancer (TNBC) is defined as any breast cancer that does not express the genes for estrogen receptor (ER), progesterone receptor (PR) and human epidermal growth factor receptor 2 (HER-2). It accounts for 15-20% of breast cancer cases, representing ~40,000 new diagnoses each year in the United States [1]. The TNBC phenotype is manifested in 48-70% of breast cancers with the breast cancer (BRCA) gene 1 mutation; however, this phenotype is sporadic and predominantly lacks BRCA mutations [2, 3]. Even though TNBC and the basal subtype often are referred to interchangeably based on complementary deoxyribonucleic acid (cDNA) tissue microarray analysis, there exists about 15-30% discordance between these cancers [4, 5]. At a molecular level, TNBC is an extremely heterogeneous disease [6].

The genomics, natural history and clinical course of TNBC have not been well studied, compared with the better known hormone receptor positive subtypes. TNBC is more sensitive to cytotoxic chemotherapy than receptor positive luminal A breast cancer, yet the prognosis is significantly inferior for this subtype [7]. Currently, no targeted therapy is available for TNBC, owing to the lack of an identifiable receptor.

Earlier trials for resectable breast cancer did not show improved overall (OS) or disease-free (DFS) survival when comparing neoadjuvant (NACT) and adjuvant (ACT) chemotherapy [8–13]. A recent updated report showed that the addition of Taxane and Adriamycin-cyclophosphamide preoperatively improved the pathological complete response (pCR) rate, but no difference was observed for OS or DFS [8]. However, this result contradicts earlier generation studies that showed increased OS and DFS with pCR [14, 15]. These trials were conducted at a time when the molecular heterogeneity of breast cancer was less understood; therefore, it is not well established whether or not NACT provides a superior outcome based on different breast cancer subtypes included in this TNBC cohort.

Subsequent to the above-mentioned trials, several reports regarding TNBC subtype have indicated improved outcomes when pCR is achieved following NACT. These results are comparable with the favorable results observed for luminal A breast cancer. Accordingly, pCR may serve as a suitable surrogate end point when assessing the treatment benefit of the TNBC patient population [16, 17].

In this study of patients with stage I-III TNBC, the aim was to assess the impact of treatment and tumor-related factors on patient outcomes (OS and DFS).

## RESULTS

Patients were aged from 21-88 years and were followed for a median of 3.9 years (range=0.14-14) (Table 1). Almost half (48%) of the cohort was black, 51% presented with stage II disease, and 87% had grade 3 tumors. The most commonly used chemotherapy components were anthracycline (63%) or taxane (73%) (Table 2). A small number of patients received other combination chemotherapy including cyclophosphamide, methotrexate and 5 fluorouracil or platinum based regimens.

**Table 1:** Patient characteristics (n= 452)

Characteristic	n (%)
**Demographic/Patient Factors**	
Age (Years)	
≤40	92 (20)
41-60	262 (58)
61+	98 (22)
Median [Range]	51 [21−88]
Race	
White	216 (48)
Black	214 (47)
Hispanic/Other	22 (5)
Grade	
Well/Moderately differentiated (1/2)	50 (11)
Poorly differentiated (3)	379 (84)
Unknown	23 (5)
Stage	
1	91 (20)
2	230 (51)
3	131 (29)
LVI	
Absent	299 (66)
Present	131 (28)
Not reported/unknown	35 (6)
Year period	
≤2005	179 (40)
>2005	273 (60)

LVI=lymphovascular invasion.

**Table 2:** Treatment characteristics (n=452)

Characteristic	n (%)
Surgery	
None	16 (4)
BCS	210 (46)
Mastectomy	226 (50)
Chemotherapy	
None	32 (7)
NACT, pCR	67 (15)
NACT, RD	135 (30)
ACT	218 (48)
Health insurance type	
Medicaid	53 (12)
Medicare	46 (10)
Medicare+supplement	78 (17)
Military	15 (3)
No Insurance	80 (18)
Private	180 (40)
NACT	
pCR	67 (33)
RD	135 (67)
Radiation	
Yes	335 (74)
No	117 (26)
T-AC/T	
Yes	286 (63)
No	166 (37)

ACT=adjuvant chemotherapy, BCS= breast conserving surgery, NACT=neoadjuvant chemotherapy, pCR=pathological complete response, RD=residual disease, T-AC/T=Adriamycin/Cyclophasphomide+Taxol/Taxane.

Surgery, consisting of either breast conserving surgery (BCS) or mastectomy, was associated with a significant OS (*P*<0.0001) and DFS (*P*<0.0001) benefit. Patients with stage III tumors (OS, *P*<0.0001; DFS, *P*<0.0001), lymphovascular invasion (LVI) (OS, *P*<0.039; DFS, *P*<0.027) or Medicaid health insurance (OS, *P*<0.0007; DFS, *P*<0.0001) had inferior survival outcomes. Having no health insurance was significant for OS (*P*=0.031); but not for DFS (*P*=0.065). Age, grade, race, radiation therapy, and year of treatment were not significant predictors of either OS or DFS.

### Effect of timing of chemotherapy and response rate

**Figure 1:**
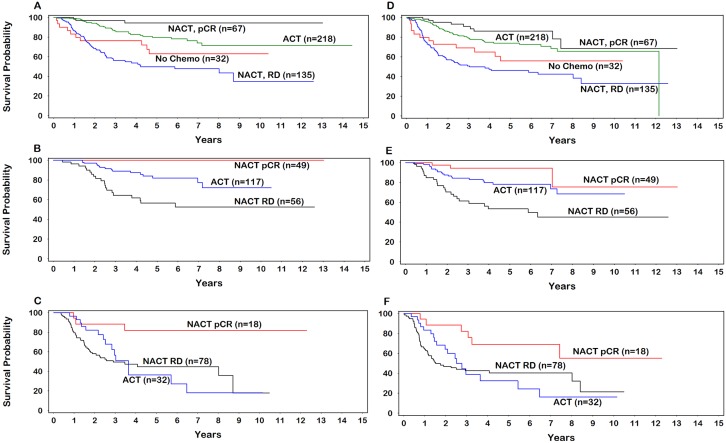
Overall survival (**A.** all patients; **B.** stage II; **C.** stage III), Disease-free survival (**D.** all patients; **E.** stage II; **F.** stage III). ACT=adjuvant chemotherapy; NACT=neoadjuvant chemotherapy; pCR=pathological complete response; RD=residual disease.

Approximately 33% of patients achieved pCR following NACT (n=202) whereas 67% had RD either in the breast or in the lymph nodes (Table 2). Compared with patients receiving no chemotherapy, patients achieving pCR following NACT had significantly better OS (*P*=0.0024) and DFS (*P*=0.0080) (Figure 1A and 1D). Both OS (*P*=0.052) and DFS (*P*=0.075) were not statistically significant for patients receiving ACT. Additionally, both ACT (*P*<0.0001) and pCR following NACT (*P*<0.0001), were significantly associated with improved OS and DFS, compared with RD following NACT.

### Effect of response to chemotherapy on different stage

The timing of chemotherapy by stage and type of response to NACT was assessed as possible correlates of OS and DFS (Table 3). As expected, in stage I disease, 99% of patients received ACT compared with stage III disease where 75% of patients received NACT. In stage II, 53% of patients received ACT while 47% received NACT. Among patients receiving NACT, 47% achieved pCR in stage II disease versus 19% of those with stage III disease.

**Table 3:** Response to chemotherapy by stage (n=452)

Characteristic	n (%)
Stage I	
NACT	1 (1)
ACT	69 (99)
Stage II	
NACT, RD	56 (25)
NACT, pCR	49 (22)
ACT	117 (53)
Stage II (NACT only)	
RD	56 (53)
pCR	49 (47)
Stage III	
NACT, RD	78 (61)
NACT, pCR	18 (14)
ACT	32 (25)
Stage III (NACT only)	
RD	78 (81)
pCR	18 (19)

ACT=adjuvant chemotherapy, NACT=neoadjuvant chemotherapy, pCR=pathological complete response, RD=residual disease.

Among patients with stage II disease, both pCR following NACT and ACT were associated with significantly improved OS (HR undefined, score statistic p<0.0001; HR 0.35, 95%CI=0.18-0.67) and DFS (HR 0.14, 95%CI=0.04-0.45; HR 0.39, 95%CI=0.22-0.70) compared with the RD group (Figure 1B and 1E). In contrast, only pCR following NACT was a statistically significant predictor of improved OS (HR 0.19, 95%CI=0.06-0.63) and DFS (HR 0.35, 95%CI=0.15-0.82) among patients with stage III disease (Figure 1C and 1F). OS and DFS were not significantly associated with the use of ACT among patients with stage III disease.

### Multivariable analysis

BCS (*P*=0.0006), mastectomy (*P*<0.0001), stage (*P*<0.0001), ACT (*P*=0.0017) and pCR following NACT (*P*<0.0001) were observed to be positive independent predictors of OS in a multivariable analysis (MVA). Similarly, BCS (*P*=0.020), mastectomy (*P*<0.0001), ACT (*P*=0.050), pCR after NACT (*P*<0.0019), and early stage (*P*<0.0001) were observed to be positive independent predictors of DFS. In contrast, Medicaid health insurance was a significant negative independent predictor of both OS (*P*=0.017) and DFS (*P*=0.0014) compared with other health insurance types. Analysis showed that age, grade, LVI, race, radiotherapy, and year of treatment were not statistically significant predictors of either OS or DFS.

## DISCUSSION

In this analysis of TNBC, we comprehensively examined patient demographics, treatment factors, and the effect of timing and response to chemotherapy on survival outcomes, based on different stages of tumor.

Traditionally, NACT has been an acceptable option among patients who are candidates for chemotherapy and prefer organ preservation, based on the National Surgical Adjuvant Breast and Bowel Project (NSABP B-18 and B-27) randomized studies comparing NACT with ACT [8, 9]. Recent studies have shown that while TNBC had higher response rates to chemotherapy compared with hormone receptor positive subgroups, this has not translated into superior survival outcomes [6, 7]. It also has been established for certain aggressive subtypes of breast cancer, including HER-2 (over-expressed) and TNBC, that achieving pCR provides an important surrogate marker for predicting long term clinical response and survival outcomes [16, 17].

The results of a large meta-analysis [16] of 6,377 patients with operable or advanced non-metastatic disease from 6 prospective neoadjuvant studies, suggested that pCR is an effective surrogate marker for survival among patients with luminal-B/HER-2 negative, HER-2 positive, and TNBC tumors. The prognostic impact of pCR was observed to be greatest among patients with HER-2 positive, and TNBC. In the TNBC group of 911 patients who received anthracycline and taxane based NACT, 31% achieved pCR. There was no difference in OS or DFS among patients who achieved pCR.

In a related study, a statistically significant survival benefit was observed when pCR was achieved following NACT among patients with TNBC. This was equivalent to the non-TNBC group experiencing pCR [17]. When RD was present following NACT, the survival outcome was significantly inferior to the non-TNBC group. Furthermore, 22% of patients in the TNBC group experienced pCR compared with 11% in the non-TNBC group, indicating a superior or similar responsiveness to chemotherapy [17].

In our study, we observed a significant OS and DFS benefit among TNBC patients receiving any systemic therapy/NACT versus no chemotherapy in terms of pCR. The rate of pCR (33%) was similar to that in the literature reported here.

The NSABP B-18 and B-27 clinical trial series has not shown any survival advantage of using NACT over ACT [8, 9]. These studies were performed at a time when breast cancer was not differentiated into subtypes based on their receptor expression and so, it remains unknown whether or not a survival benefit exists for particular subtypes of breast cancers, given their reported heterogeneity. In an attempt to answer this question, a retrospective study of 493 patients with TNBC was conducted to compare outcomes among patients receiving NACT versus ACT [18]. Researchers noted that patients receiving ACT were less likely to die of their disease than patients receiving NACT. This study was limited by a lack of information on the pCR rate following NACT. Based on available data, achieving pCR appears to be a key predictor of improved outcomes in TNBC. Questions remain on how to best integrate systemic therapy, given the increased risk of early dissemination for this disease.

A similar OS benefit was observed in our study among patients who achieved pCR with NACT compared with those who experienced RD. For stage III disease, achieving pCR following NACT was a significant predictor for both OS and DFS. In this subgroup, ACT was significantly inferior to patients achieving pCR following initial chemotherapy.

A comparison of pCR rates for operable, locally advanced and inflammatory breast cancer following NACT was assessed in the GeparTrio trial [19]. In univariable analysis, the pCR rate was significantly lower in the inflammatory (8.6%) and advanced groups (11.3%), compared with the operable group (17.7%) (p=0.002). This study did not analyze the impact of pCR on survival among patients with more advanced disease nor did it address different breast cancer subtypes. This study does indicates a lower rate of pCR among patients with more advanced stage disease across the spectrum of breast cancer subtypes. Similarly, we observed a differential response rate of TNBC to NACT based on tumor stage, with less pCR observed among patients with advanced stage cancer. For patients with stage II disease, the pCR rate was 53% versus 19% for stage III disease.

Our study showed a decreasing pCR rate following chemotherapy with advancing tumor stage, which correlated with inferior patient survival. The results are unique as the analysis was limited to TNBC subtype and confirmed the importance of achieving pCR. Our results suggest that with advancing stage, the rate of pCR was lowered proportionately, which may indicate an increase of chemotherapy resistant clones as the tumor grows in size.

Racial and ethnic disparities are widely believed to exist for breast cancer outcomes, with black women reported to have poorer survival than white women [20, 21]. In contrast, no differences were reported in pCR rates, relapse-free survival or OS for TNBC between black and white patients [22]. Our study, which included a large number of black women with TNBC, reported no differences in OS or DFS across race for either univariable or multivariable analysis.

Socioeconomic deprivation is believed to be an important predictive factor of increased mortality, among black and Hispanic women and, to a lesser degree, among white women [23, 24]. With lung cancer, having Medicaid or having no health insurance have been associated with poor survival outcomes [25]. More recently, based on a SEER database analysis, a similar result was reported for breast cancer and 9 other cancers in the United States. Patients having Medicaid or no health insurance had significantly worse outcomes than patients having other health insurance types [26]. We found that having Medicaid health insurance was a significant differential predictor for shorter survival, even after adjusting for several outcome-related covariates.

This study has some limitations. Information was not available for patient income, so it is unknown, whether or not health insurance status was an accurate and reliable surrogate measure for socioeconomic status [27]. This study's observational design led to potential selection bias. Despite this, various demographic and treatment-related factors were included in the MVA to adjust for potential confounding that may have influenced results. The study was strengthen by its large sample size and a high percentage of black patients. Furthermore, differential outcomes were uniquely examined based on the timing of chemotherapy, response rates, and stage of disease.

We conclude that patients who achieved pCR following NACT had superior survival outcomes compared with other patients in this TNBC study cohort. Important differences were observed when our results were stratified by stage. Both superior OS and DFS were seen for patients with stage III disease who experienced pCR following NACT. In contrast, for patients with stage II disease, DFS rates were similar for ACT and pCR following NACT. The adjustment for other outcome related covariates did not significantly alter the findings. These results suggest that novel strategies are needed to improve the rate of pCR in advanced TNBC. More research is needed to differentiate responders to chemotherapy from those who are unlikely to achieve pCR.

## MATERIALS AND METHODS

A total of 452 tumor registry patients (Central/Eastern North Carolina) with negative ER/PR and HER-2 receptor status and no evidence of metastatic disease were identified over the period of 1996-2011. All cancers were pathologically confirmed. ER or PR was considered negative if the expression was ≤ 1% based on immunohistochemistry (IHC). HER-2 was considered non-expressive based on IHC (0 or 1+) and/or fluorescence *in situ* hybridization (FISH). Tumor characteristics and patient information were collected from the cancer registry and electronic medical records. Stage was based on pre-treatment clinical and imaging information. Staging was performed according to the American Joint Committee on Cancer (AJCC) – 6^th^ edition guidelines. Grade was based on the modified Nottingham histologic score and divided into grade 1, 2 or 3 (well, moderately, or poorly differentiated). Information on the presence of LVI, treatment parameters (surgery, radiotherapy, chemotherapy) and health insurance status were collected. Surgical treatment was categorized as BCS or mastectomy. Chemotherapy included NACT or ACT. After NACT, pCR was achieved if there was no invasive cancer detected in surgical specimens from breast or lymph nodes. Any invasive cancer in breast or lymph nodes was scored as residual disease (RD). This study received institutional review board approval.

### Statistical analysis

Parameters collected were age at diagnosis, grade, histology, LVI, NACT or ACT, pCR following NACT, race, radiotherapy, surgical management, tumor and nodal stage at diagnosis, and type of health insurance.

OS time was computed from the date of diagnosis until death or last follow-up. DFS was computed from the date of diagnosis until the date of any failure. Patients were censored if they did not have any event on their last follow-up date. The Kaplan-Meier method was used to estimate OS and DFS. A proportional-hazards regression model was used to estimate hazard ratios (HRs), *P*-values, and 95% confidence intervals (CIs) for survival time differences. *P*-values were computed using the score statistic when parameter convergence was not achieved (e.g., no events in one of the comparison groups). All reported *P*-values were two-sided. The statistical significance level was set at *P*<0.05. Statistical analysis was performed using SAS v9.4 (Cary, NC). The proportional-hazards assumption was not violated in our analysis [28].

## Abbreviations

ACT: Adjuvant chemotherapy
AJCC: American Joint Committee on Cancer
BCS: Breast conserving surgery
BRCA: Breast cancer
cDNA: Complementary deoxyribonucleic acid
DFS: Disease-free survival
ER: Estrogen receptor
FISH: Fluorescence *in situ* hybridization
HER 2: Human epidermal growth factor receptor 2
HR: Hazard ratio
IHC: Immunohistochemistry
LVI: Lymphovascular invasion
MVA: Multivariable analysis
NACT: Neoadjuvant chemotherapy
NSABP: National Surgical Adjuvant Breast and Bowel Project
pCR: Pathological complete response
PR: Progesterone receptor
RD: Residual disease
TNBC: Triple negative breast cancer
OS: Overall survival

## References

[1] Silver DP, Richardson AL, Eklund AC, Wang ZC, Szallasi Z, Li Q, Juul N, Leong CO, Calogrias D, Buraimoh A, Fatima A, Gelman RS, Ryan PD (2010). Efficacy of neoadjuvant Cisplatin in triple-negative breast cancer. J Clin Oncol.

[2] Lee E, McKean-Cowdin R, Ma H, Spicer DV, Van Den Berg D, Bernstein L, Ursin G (2011). Characteristics of triple-negative breast cancer in patients with a BRCA1 mutation: results from a population-based study of young women. J Clin Oncol.

[3] Atchley DP, Albarracin CT, Lopez A, Valero V, Amos CI, Gonzalez-Angulo AM, Hortobagyi GN, Arun BK (2008). Clinical and pathologic characteristics of patients with BRCA-positive and BRCA-negative breast cancer. J Clin Oncol.

[4] Foulkes WD, Smith IE, Reis-Filho JS (2010). Triple-negative breast cancer. N Engl J Med.

[5] Anders CK, Carey LA (2009). Biology, metastatic patterns, and treatment of patients with triple-negative breast cancer. Clin Breast Cancer.

[6] Rouzier R, Perou CM, Symmans WF, Ibrahim N, Cristofanilli M, Anderson K, Hess KR, Stec J, Ayers M, Wagner P, Morandi P, Fan C, Rabiul I (2005). Breast cancer molecular subtypes respond differently to preoperative chemotherapy. Clin Cancer Res.

[7] Carey LA, Dees EC, Sawyer L, Gatti L, Moore DT, Collichio F, Ollila DW, Sartor CI, Graham ML, Perou CM (2007). The triple negative paradox: primary tumor chemosensitivity of breast cancer subtypes. Clin Cancer Res.

[8] Rastogi P, Anderson SJ, Bear HD, Geyer CE, Kahlenberg MS, Robidoux A, Margolese RG, Hoehn JL, Vogel VG, Dakhil SR, Tamkus D, King KM, Pajon ER (2008). Preoperative chemotherapy: updates of National Surgical Adjuvant Breast and Bowel Project Protocols B-18 and B-27. J Clin Oncol.

[9] Wolmark N, Wang J, Mamounas E, Bryant J, Fisher B (2001). Preoperative chemotherapy in patients with operable breast cancer: nine-year results from National Surgical Adjuvant Breast and Bowel Project B-18. J Natl Cancer Inst Monogr.

[10] Bonadonna G, Veronesi U, Brambilla C, Ferrari L, Luini A, Greco M, Bartoli C, Coopmans de Yoldi G, Zucali R, Rilke F, Andreola S, Silvestrini R, Di Fronzo G (1990). Primary chemotherapy to avoid mastectomy in tumors with diameters of three centimeters or more. J Natl Cancer Inst.

[11] Hortobagyi GN, Blumenschein GR, Spanos W, Montague ED, Buzdar AU, Yap HY, Schell F (1983). Multimodal treatment of locoregionally advanced breast cancer. Cancer.

[12] Hortobagyi GN, Ames FC, Buzdar AU, Kau SW, McNeese MD, Paulus D, Hug V, Holmes FA, Romsdahl MM, Fraschini G (1988). Management of stage III primary breast cancer with primary chemotherapy, surgery, and radiation therapy. Cancer.

[13] Hortobagyi GN (1990). Comprehensive management of locally advanced breast cancer. Cancer.

[14] Bonadonna G, Valagussa P, Brambilla C, Ferrari L, Moliterni A, Terenziani M, Zambetti M (1998). Primary chemotherapy in operable breast cancer: eight-year experience at the Milan Cancer Institute. J Clin Oncol.

[15] Fisher B, Bryant J, Wolmark N, Mamounas E, Brown A, Fisher ER, Wickerham DL, Begovic M, DeCillis A, Robidoux A, Margolese RG, Cruz AB, Hoehn JL (1998). Effect of preoperative chemotherapy on the outcome of women with operable breast cancer. J Clin Oncol.

[16] von Minckwitz G, Untch M, Blohmer JU, Costa SD, Eidtmann H, Fasching PA, Gerber B, Eiermann W, Hilfrich J, Huober J, Jackisch C, Kaufmann M, Konecny GE (2012). Definition and impact of pathologic complete response on prognosis after neoadjuvant chemotherapy in various intrinsic breast cancer subtypes. J Clin Oncol.

[17] Liedtke C, Mazouni C, Hess KR, Andre F, Tordai A, Mejia JA, Symmans WF, Gonzalez-Angulo AM, Hennessy B, Green M, Cristofanilli M, Hortobagyi GN, Pusztai L (2008). Response to neoadjuvant therapy and long-term survival in patients with triple-negative breast cancer. J Clin Oncol.

[18] Kennedy CR, Gao F, Margenthaler JA (2010). Neoadjuvant versus adjuvant chemotherapy for triple negative breast cancer. J Surg Res.

[19] Costa SD, Loibl S, Kaufmann M, Zahm DM, Hilfrich J, Huober J, Eidtmann H, du Bois A, Blohmer JU, Ataseven B, Weiss E, Tesch H, Gerber B (2010). Neoadjuvant chemotherapy shows similar response in patients with inflammatory or locally advanced breast cancer when compared with operable breast cancer: a secondary analysis of the GeparTrio trial data. J Clin Oncol.

[20] Weir HK, Thun MJ, Hankey BF, Ries LA, Howe HL, Wingo PA, Jemal A, Ward E, Anderson RN, Edwards BK (2003). Annual report to the nation on the status of cancer, 1975-2000, featuring the uses of surveillance data for cancer prevention and control. J Natl Cancer Inst.

[21] Sachdev JC, Ahmed S, Mirza MM, Farooq A, Kronish L, Jahanzeb M (2010). Does race affect outcomes in triple negative breast cancer?. Breast Cancer (Auckl).

[22] Dawood S, Broglio K, Kau SW, Green MC, Giordano SH, Meric-Bernstam F, Buchholz TA, Albarracin C, Yang WT, Hennessy BT, Hortobagyi GN, Gonzalez-Angulo AM (2009). Triple receptor-negative breast cancer: the effect of race on response to primary systemic treatment and survival outcomes. J Clin Oncol.

[23] Vona-Davis L, Rose DP (2009). The influence of socioeconomic disparities on breast cancer tumor biology and prognosis: a review. J Womens Health (Larchmt).

[24] Thomson CS, Hole DJ, Twelves CJ, Brewster DH, Black RJ (2001). Prognostic factors in women with breast cancer: distribution by socioeconomic status and effect on differences in survival. J Epidemiol Community Health.

[25] Slatore CG, Au DH, Gould MK (2010). An official American Thoracic Society systematic review: insurance status and disparities in lung cancer practices and outcomes. Am J Respir Crit Care Med.

[26] Walker GV, Grant SR, Guadagnolo BA, Hoffman KE, Smith BD, Koshy M, Allen PK, Mahmood U (2014). Disparities in stage at diagnosis, treatment, and survival in nonelderly adult patients with cancer according to insurance status. J Clin Oncol.

[27] Ovcaricek T, Frkovic SG, Matos E, Mozina B, Borstnar S (2011). Triple negative breast cancer – prognostic factors and survival. Radiol Oncol.

[28] Grambsch PM, Therneau TM (1994). Proportional hazards tests and diagnostics based on weighted residuals. Biometrika.

